# Supplementary material for: Using Ex Vivo Porcine Jejunum to Identify Membrane Transporter Substrates: A Screening Tool for Early—Stage Drug Development
Source: Biomedicines. 2020 Sep 10;8(9):340. doi: 10.3390/biomedicines8090340 (PMC7555276; doi:10.3390/biomedicines8090340)
Supplement: Supplementary file 1 [file biomedicines-08-00340-s001.pdf]

biomedicines

Supplementary Material:

Using ex vivo porcine jejunum to identify membrane transporter substrates: a screening tool for early-stage drug development

Yvonne E. Arnold[1,2] and Yogeshvar N. Kalia[1,2]

1. School of Pharmaceutical Sciences, University of Geneva, CMU - 1 rue Michel Servet, 1211 Geneva 4, Switzerland
2. Institute of Pharmaceutical Sciences of Western Switzerland, University of Geneva, CMU - 1 rue Michel Servet, 1211 Geneva 4, Switzerland

Table S1. UHPLC-MS/MS methods.

Compound	Chromatographic Conditions					Mass Spectrometry Conditions					
	Mobile Phases		Col. Temp. (°C)	Flow Rate (mL/min)	Retention Time (min)	Capillary Voltage (kV)	Cone Voltage (V)	Collision Energy (eV)	MRM Transition (m/z)	ESI	LOQ (nM)
	Aqueous	Organic									
Cefadroxil	50% A	50% C	40	0.2	0.79	2.7	8	6	364.04 > 208.03	+ve	0.38
Rosuvastatin	50% A	50% D	40	0.2	1.30	2.6	14	28	482.12 > 258.09	+ve	1.00
Ranitidine	30% A	70% C	45	0.2	0.83	2.4	6	20	315.14 > 97.82	+ve	2.88
Digoxin	50% A	50% C	35	0.2	0.82	2.9	70	20	779.41 > 649.62	-ve	0.75
Sulfasalazine	50% A	50% C	40	0.2	0.97	2.8	2	26	398.98 > 223.02	+ve	1.18
Valsartan	20% B	80% C	40	0.2	0.77	2.5	18	14	436.16 > 291.28	+ve	2.72
Fexofenadine	20% A	80% G	40	0.2	0.92	2.1	60	24	502.38 > 466.35	+ve	1.63

Aqueous phases: A: 0.1% formic acid in H_2O
B: 5mM ammonium formate in H_2O, pH = 3.5

Organic phases: C: 0.1% formic acid in acetonitrile
D: acetonitrile

Table S2. The precision and accuracy of the analytical methods.

Compound	Theoretical Concentration (nM)	Experimental Concentration (nM)	Precision (%)	Accuracy (%)
	78.43	82.93 ± 1.75	2.11	105.74
Cefadroxil	235.29	225.46 ± 8.87	3.94	95.83
	588.21	612.78 ± 10.87	1.77	104.18
	77.88	78.49 ± 0.88	1.12	100.79
Rosuvastatin	103.83	95.03 ± 6.53	6.88	91.52
	181.71	182.71 ± 2.89	1.58	100.55
	26.24	24.99 ± 0.39	1.55	95.25
Ranitidine	262.40	265.99 ± 5.87	2.21	101.37
	524.81	544.45 ± 9.91	1.82	103.74
	44.10	48.57 ± 0.12	0.25	110.14
Digoxin	222.10	229.91 ± 14.47	6.29	103.52
	444.18	447.43 ± 22.16	4.66	107.05
	15.06	15.14 1.52	10.29	100.52
Sulfasalazine	75.30	68.04 ± 3.74	5.49	90.36
	150.60	144.77 ± 4.45	2.95	100.30
	106.20	108.43 ± 3.54	3.26	102.11
Valsartan	212.39	218.63 ± 19.37	8.86	102.94
	2123.90	2398.72 ± 137.52	5.73	112.94
	14.45	14.59 ± 1.46	9.99	100.95
Fexofenadine	57.81	61.22 ± 4.93	8.06	105.91
	144.51	156.46 ± 11.18	7.15	108.26
